# OA18 Navigating complexities in male paediatric-onset lupus: From macrophage activation syndrome and refractory lupus nephritis to CAR-T cell therapy

**DOI:** 10.1093/rap/rkae117.018

**Published:** 2024-11-05

**Authors:** Anastasia Vasiliki Madenidou, Ben Parker

**Affiliations:** Centre for Musculoskeletal Research, The University of Manchester, Manchester, United Kingdom; The Kellgren Centre for Rheumatology, Manchester University Hospitals NHS Foundation Trust, Manchester, United Kingdom; The Kellgren Centre for Rheumatology, Manchester University Hospitals NHS Foundation Trust, Manchester, United Kingdom

## Abstract

**Introduction:**

Although systemic lupus erythematosus (SLE) is less common in male than female population, male patients usually present with more severe manifestations. Patients with lupus, especially the ones with childhood-onset disease, may try multiple immunosuppresive medications over the years with partial response. Here, we present a male patient diagnosed with SLE at the age of 10, who after having multiple-treatments under the care of paediatric and adult rheumatolgy, is being screened for chimeric antigen receptor (CAR) -T cell therapy for refractory lupus nephritis (phase I trial).

**Case description:**

A male patient was diagnosed with SLE at the age of 10 when presented with fever, arthritis and macrophage activation syndrome (MAS). His serology included low complement, and positive ANA, dsDNA, anti-chromatin, ant-ribosomal P, and Ro antibodies. In 2017, he developed muscle weakness and had a muscle biopsy showing necrotic myopathy. He had multiple medications over the years, which were not well-tolerated: mycophenolate mophetil (gastrointestinal disturbance), azathioprine (low white cell count), and hydroxychloroquine (significant diarroea). After being seen in the transition rheumatology clinic, he transitioned to the adult service in 2021. In the same year, while being on methotrexate, he developed class IV lupus nephritis (LN). Subsequently, he had cyclophsophamide (EuroLupus regimen) followed by mycophenolic acid as maintenance treatment. Rituximab was added in 2021 because of partial renal respone [urine protein/creatinine (PCR) ratio at 186 mg/dl]. Voclosporin was added at the end of 2023 with no significant improvement. In May 2024, the patient was on mycophenolic acid 180 mg bd (maximum tolerated dose), Prednisolone 10 mg od, and Voclosporin 23.7 mg twice a day, and had 6-monthly Rituximab (last dose in February 2024). Despite the combination treatment, he still had persistent proteinuria (urine PCR at 232 mg/dl) with no extra-renal manifestations and experienced difficulties in taking many tablets. His kidney function was preserved (estimated glomerural filtration rate at 90 ml/min), dsDNA levels were raised at 34 IU/ml and C4 was low at 0.09 g/L. A repeat kidney biopsy has been booked for 8th July to ensure there is still active LN and discussions about further treatment started. Adding immunosuppresive medications, such as belimumab, or trying CAR-T cell therapy (phase I for LN) was offered. The patient decided to explore CAR-T cell therapy, has already had the first haematology consultation, and screening has been booked for early July.

**Discussion:**

This case demonstrates the challenges we experience in rheumatology when dealing with complex lupus cases. It is also not a surprise that our patient is male and has childhood-onset disease. Childhood onset lupus is often more severe, even when they are in adult services. Younger patients can also be very refractory. Male lupus and childhood onset may have a significant genetic basis, but doesn’t appear to be a clear genetic cause in this case. Although our patient had multiple medications over the years, including new ones, such as voclosporin, his LN is still not in remission. Despite the importance of clinical guidelines, sometimes we run out of options and we need to explore other treatments, such as participation in clinical trials. It is also vital to discuss all treatment options with our patients, in our case trying another immunosuppressive treatment or participating in a clinical trial. With this case, we would like to raise awareness that participation in clinical trials should be explored as a treatment option, especially for patients with refractory disease. We are hoping to present this case at the Case-based conference to discuss the patient’s journey from childhood till today and we are looking forward to hearing the thoughts from the adult and paediatric rheumatology audience. We would also like to explore if the audience would consider other treatment options, including CAR-T cell therapy. We are also planning to present the local CAR-T cell therapy set-up which involves joint care between rheumatology and haematology and raise awareness about this new treatment option which promises drug-free remission. We would also like to emphasise that timing of CAR-T cell therapy is critical, ideally before accumulation of significant damage.

**Key learning points:**

• One of the key learning points is that lupus can present in male patients and can have severe manifestations, such as lupus nephritis. Following a patient’s during childhood, you see the differences between paediatric and adult rheumatology, such as presentation of lupus with MAS in childhood. This is especially important for the patients we see around the age of 16 years old who may present with symptoms more suggestive of paediatric rather than adult lupus. Smooth trasition from paediatric to adult service is a key step for successful management of patients with childhood-onset diseases. Clinical trials offer additional treatment options, especially in cases that we have run out of options. Share decision making and offering opportunities that align with patients’ values, such as the potential of drug-free remission with CAR-T cell therapy, are paramount. During the conference, we are looking forward to hearing from both paediatric and adult colleagues how they would manage this patient. We are also hoping the patient to have started on CAR-T cell therapy by the start of the conference, so we can present our experience with CAR-T cell therapy and in any case, to raise awareness about this new novel treatment which has the potential to revolutianise the treatment of rheumatic diseases.

